# Differential Expression of the Host Lipid Regulators ANGPTL-3 and ANGPTL-4 in HCV Infection and Treatment

**DOI:** 10.3390/ijms22157961

**Published:** 2021-07-26

**Authors:** Vaia Valiakou, Petros Eliadis, Eirini Karamichali, Ourania Tsitsilonis, John Koskinas, Urania Georgopoulou, Pelagia Foka

**Affiliations:** 1 Molecular Virology Laboratory, Hellenic Pasteur Institute, 11521 Athens, Greece; vaiavaliakou@pasteur.gr (V.V.); eirinik@pasteur.gr (E.K.); 2 Molecular Biology and Immunobiotechnology Laboratory, Hellenic Pasteur Institute, 11521 Athens, Greece; peliadis@pasteur.gr; 3 Department of Biology, National and Kapodistrian University of Athens (NKUA), 15784 Athens, Greece; rtsitsil@biol.uoa.gr; 4 2nd Department of Internal Medicine, Medical School of Athens, Hippokration Hospital, 11521 Athens, Greece; koskinasj@yahoo.gr

**Keywords:** angiopoietin-like proteins, HCV infection, fingerprint, lipid metabolism, DAA, liver, cirrhosis, fibrosis, TGF-β, hepatocellular carcinoma

## Abstract

Host lipid metabolism reprogramming is essential for hepatitis C virus (HCV) infection and progression to severe liver disease. Direct-acting antivirals (DAAs) achieve a sustained virological response (SVR) in most patients, but virus eradication does not always protect against hepatocellular carcinoma (HCC). Angiopoietin-like protein-3 (ANGPTL-3) and angiopoietin-like protein-4 (ANGPTL-4) regulate the clearance of plasma lipids by inhibiting cellular lipase activity and possess emerging roles in tumourigenesis. We used ELISA and RT-qPCR to investigate ANGPTL-3 and ANGPTL-4 expression in HCV patients with characterised fibrosis throughout the natural history of hepatitis C and in long-term HCV infection in vitro, before and after DAA treatment. ANGPTL-3 was decreased in patients with advanced fibrosis compared to other disease stages, while ANGPTL-4 was progressively increased from acute infection to cirrhosis and HCC, peaking at the advanced fibrosis stage. Only ANGPTL-3 mRNA was down-regulated during early infection in vitro, although both ANGPTLs were increased later. DAA treatment did not alter ANGPTL-3 levels in advanced fibrosis/cirrhosis and in HCV infection in vitro, in contrast to ANGPTL-4. The association between ANGPTLs and fibrosis in HCV infection was underlined by an inverse correlation between the levels of ANGPTLs and serum transforming growth factor- β (TGF-β). Collectively, we demonstrate the pivotal role of advanced fibrosis in defining the expression fate of ANGPTLs in HCV infection and after treatment and propose a role for ANGPTL-3 as a contributor to post-treatment deregulation of lipid metabolism that could predispose certain individuals to HCC development.

## 1. Introduction

Hepatitis C is a viral disease with a global geographical distribution, with approximately 71,000,000 people chronically infected worldwide [1]. Chronicity is established in 60–80% of hepatitis C virus (HCV)-infected individuals, accompanied by an increased risk for cirrhosis development within the next 20 years. At least 5–7% of cirrhotic patients develop hepatocellular carcinoma (HCC) annually [2,3].

The mostly asymptomatic nature of the infection during the early years enables the virus to stealthily alter liver physiology. HCV viral proteins, mainly core and non-structural 5A (NS5A), orchestrate de novo lipogenesis and enhance hepatic lipid uptake and attenuation of fatty acid catabolism through modulation of lipid-related transcription factors, thereby promoting hepatic steatosis [4,5]. Development of hepatic fibrosis, a hallmark of HCV infection, occurs roughly at the same time. It is characterised by the excessive production of extracellular matrix (ECM) proteins from activated hepatic stellate cells (HSCs) [6]. Fibrosis comes as a result of HCV-mediated induction of fibrinogenic signalling and the secretion of profibrotic cytokines such as transforming growth factor-β (TGF-β). The molecular mechanisms that link steatosis with hepatic fibrosis are poorly defined in HCV infection. However, it has been suggested that hepatic lipid accumulation may independently cause elevated production of TGF-β by HSCs [7]. Eventually, continuous disturbance of the beneficial wound-healing processes that are part of the standard response-to-injury regeneration programme leads from set-in fibrosis to cirrhosis [8].

HCV-associated HCC occurs almost always upon a cirrhotic background. HCV causes malignant transformation of infected hepatocytes by deregulating cell proliferation, differentiation and apoptosis. Activation of oncogenic signalling, chromatin remodelling and rogue epigenetic modifications, production of oxidative stress [9,10,11] and the promotion of immunotolerance [12,13] are all important steps towards HCV-mediated tumourigenesis. The realisation that cancer cells rely mainly on de novo lipid biosynthesis for their structural, metabolic and signalling needs exposed the role of lipid metabolism in HCC development and progression [14]. Recent studies have implicated major lipid–related enzymes and specific lipid classes in hepatocarcinogenesis [15,16] while stressing the enormous impact of HCV in lipid metabolism reprogramming [17].

Since the approval of direct-acting antivirals (DAAs) in 2011, these inhibitors of NS5A, NS5B and NS3/4A HCV proteins have revolutionised HCV therapy. Cure rates have climbed up to 95% for all HCV genotypes but HCV-3a, which has proven more resistant to this type of treatment [18]. However, patients with advanced hepatic fibrosis before treatment, those with non-regressing cirrhosis, with failure in achieving sustained virological response (SVR) post-treatment, or those that display co-existing HCC-predisposing factors are still at risk for HCC [9,19,20]. Evidently, the HCV-mediated reprogramming of the host metabolism, epigenome and immunity and the constant deregulation of cellular signalling and hepatic gene expression lead to the establishment of a viral fingerprint that persistently remains after viral eradication. It is thought that these alterations may collectively promote HCC development and progression depending on the extent of the existing liver damage in some cured patients [9,10,21,22,23].

**Figure 1:**
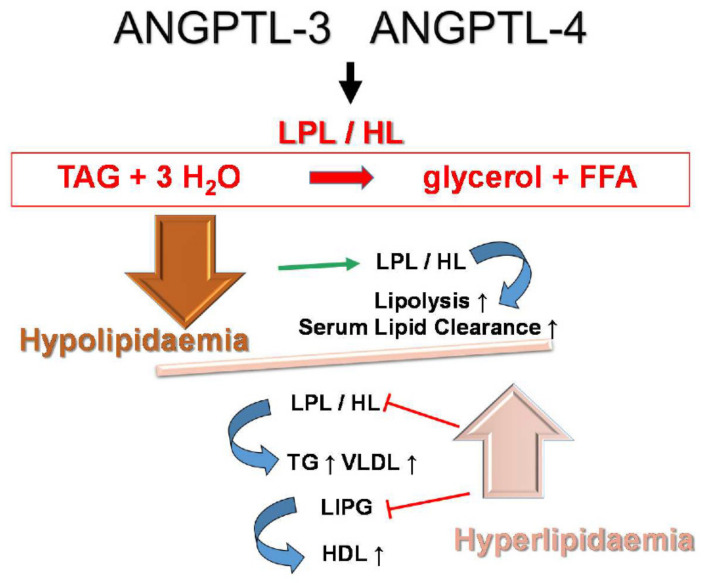
The role of angiopoietin-like proteins (ANGPTLs) in lipid metabolism. ANGPTL-3 and ANGPTL-4 regulate serum lipid levels via inhibition of the enzymatic activities of lipoprotein lipase (LPL), hepatic lipase (HL) and endothelial lipase (LIPG) (LIPG is regulated by ANGPTL-3 only). Lipases hydrolyse the triacylglycerols (TAG) of lipoproteins and chylomicrons into non-esterified free fatty acids (FFA) and monoacylglycerols. Upon silencing of ANGPTLs, loss of lipase regulation results in enhanced lipolysis and increased clearance of serum lipids by the liver and other tissues [43], eventually leading to hypolipidaemia. On the contrary, overexpression of ANGPTLs blocks lipases, thereby increasing circulating lipoproteins and resulting in hyperlipidaemia [25,26].

Angiopoietin-like proteins ANGPTL-3 and ANGPTL-4 are potent regulators of lipid metabolism (Figure 1). They both increase plasma triglycerides [24,25] through post-translational inhibition of lipoprotein lipase (LPL) and hepatic lipase (HL) enzymatic activity [26,27]. LPL hydrolyses very low density lipoproteins (VLDL) and chylomicrons into non-esterified fatty acids and monoacylglycerols, which are then absorbed for energy utilisation by peripheral tissues [28]. HL acts similarly but in the liver, where it utilises intermediate (IDL)- and high (HDL)-density lipoproteins as substrates [29]. ANGPTL-3 alone has been reported to regulate low-density lipoprotein (LDL) levels via inhibition of endothelial lipase (LIPG) activity [30]. Other known differences include tissue specificity and regulated expression, with ANGPTL-3 being mostly liver-specific and acting in an endocrine manner. ANGPTL-4 is expressed in the liver, macrophages, adipose tissue, heart and small intestine, where it locally regulates LPL-dependent lipid uptake [31,32]. On top of that, ANGPTL-3 remains largely unaffected by the dietary state, while ANGPTL-4 is strongly up-regulated by fasting [33,34]. Notably, past studies from our laboratory demonstrated that HCV core down-regulates ANGPTL-3 through cancer-related kinase minibrain-related kinase/dual-specificity tyrosine-regulated kinase 1B (Mirk/Dyrk 1B)-dependent loss of hepatic nuclear factor-1α (HNF-1α) binding activity and inhibition of liver-X/retinoid-X receptor (LXR/RXR) transactivation [35].

Independently of their role in lipid metabolism, ANGPTLs have lately been implicated in many types of cancer [36]. The exact role of ANGPTL-3 in tumour development and progression is currently unclear, as it has been shown to mediate both pro-survival [37] and antimetastatic [38] mechanisms. On the other hand, ANGPTL-4 is elevated under hypoxic and inflammatory conditions [39] and holds multiple roles as a positive regulator of angiogenesis, tumour survival and invasiveness [40,41]. Notably, we have reported that ANGPTL-3 was significantly increased in liver biopsies of patients with HCV- and hepatitis B virus (HBV)-associated HCC, compared to non-cancerous tissue [42]. Others showed that both ANGPTL-3 and ANGPTL-4 serum levels were elevated in HCV-HCC.

The scope of this study is two-fold. Firstly, we characterised the expression patterns of the host lipid regulatory factors ANGPTL-3 and ANGPTL-4 throughout the natural course of hepatitis C in vivo and HCV infection in vitro. Secondly, we assessed the input of DAA treatment, hepatic fibrosis and other clinical and viral parameters in shaping ANGPTL protein levels in HCV-induced liver disease.

## 2. Results

### 2.1. Demographical and Clinical Characteristics of Study Participants

This retrospective study was carried out using 141 sera samples collected from HCV patients with varying degrees of liver stiffness and HCV-induced HCC. The demographics and clinical characteristics of the clinical samples are presented in Table 1. The population under study was assigned to two groups, depending on whether a high or low HCV viral load (VRL) was detected at the starting time of treatment. The cut-off value of 1 × 10^6^ IU/mL was based on clinical experience and the literature [44]. Genotyping analysis revealed that HCV genotypes-1a, -1b, -2a, -3 and -4 were encountered in our samples at varied levels, except for a small percentage of samples for which there was no available record of the VRL or genotype. Depending on their clinical presentation and the METAVIR fibrosis score (Fn) recorded at the onset of therapy, the patients were categorised into the following disease stage groups: acute infection (F0/F1), mild fibrosis (F0/F1/F2), advanced fibrosis (F3), cirrhosis (F4) and HCV-induced HCC (F4).

Evidently, the numbers of samples were evenly spread across groups as far as gender, HCV VRL levels and HCV genotype were concerned, but there were significantly higher numbers of participants in the mild infection group as compared to the others (*p* < 0.005). Interestingly, we detected statistically significant differences in age, gender and serum ALT levels, but not in HCV VRL and genotype, when we performed statistical comparisons across the five disease stage groups (Table 2). Table A2 in Appendix A presents an analysis of the demographical and clinical characteristics by gender.

### 2.2. Determination of ANGPTL-3 and ANGPTL-4 Serum Levels in HCV Infection In Vivo

Early on, several studies recognised the pivotal role of liver fibrosis in the natural history of HCV infection and linked fibrosis levels to disease severity and progression [45,46]. We determined ANGPTL-3 and ANGPTL-4 levels in five groups of clinical samples ranging from the acutely infected with almost no liver fibrosis to cirrhotic patients with HCC. The use of healthy controls fell outside of the scope of this study because the objective was to assess ANGPTL expression at the different stages of HCV-induced liver disease. We measured ANGPTL-3 and ANGPTL-4 serum concentrations using the DuoSet^®^ sandwich ELISA Development kits (R&D systems, Minneapolis, MN, USA) with the modifications described in the Materials and Methods. Validation of the assays is presented in Appendix A.1.

**Figure 2:**
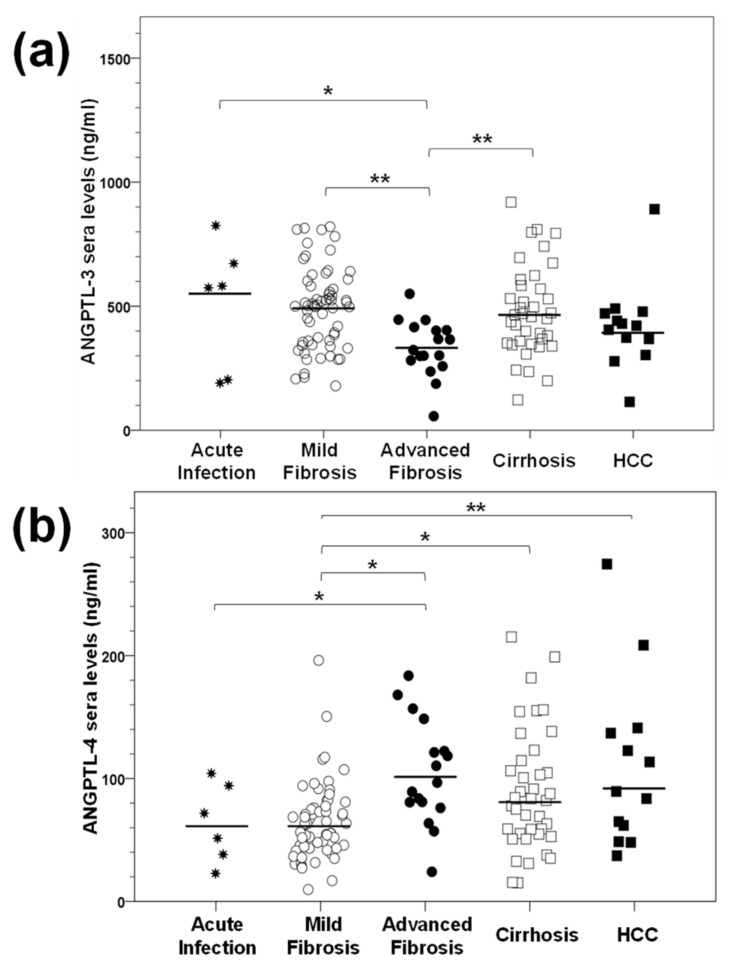
ANGPTL-3 and ANGPTL-4 serum levels throughout the natural history of HCV infection. Clinical samples of HCV patients from all stages of liver disease were used for the determination of ANGPTL-3 (**a**) and ANGPTL-4 (**b**) serum concentrations by ELISA. The black horizontal lines depict the median value of the respective group. Statistically significant differences within groups are denoted by one star (*p*-value ≤ 0.05), or two stars (*p*-value ≤ 0.005).

As shown in Table 3, there were statistically significant differences in ANGPTL-3 serum levels between the various stages of HCV-induced liver disease (*p*-value = 0.002), with the acute infection group demonstrating the highest concentration, with a median value of 563.6 ng/mL. Then, ANGPTL-3 levels kept decreasing and reached their minimum in the advanced fibrosis group (332.1 ng/mL), after which they started increasing again. Post hoc analysis revealed significant differences in the ANGPTL-3 serum levels between the pairs acute infection–advanced fibrosis (*p*-value = 0.027), mild fibrosis–advanced fibrosis (*p*-value = 0.000) and cirrhosis–advanced fibrosis (*p*-value = 0.001). We could not find any statistically significant differences within all other groups (Figure 2a).

Similar measurements were carried out for ANGPTL-4, whose serum concentration also exhibited statistically significant differences between the HCV disease stages (*p*-value = 0.002) (Table 3). However, in opposition to ANGPTL-3, ANGPTL-4 levels peaked in the advanced fibrosis group, with a value of 102.8 ng/mL, and remained relatively high in the cirrhosis and HCC groups. Notably, the lowest ANGPTL-4 concentrations were recorded in acute infection and mild fibrosis, with a median of 60.1 ng/mL. In this case, statistically significant differences were noted between the groups acute infection–advanced fibrosis (*p*-value = 0.035), mild fibrosis–advanced fibrosis (*p*-value = 0.001), mild fibrosis–cirrhosis (*p*-value = 0.007) and mild fibrosis–HCC (*p*-value = 0.021). We could not detect any statistically significant changes within all other groups (Figure 2b). Taken together, our data show a lack of a linear relationship between the expression of ANGPTLs and the time passing between acute infection and end-stage liver disease.

### 2.3. ANGPTL-3 and ANGPTL-4 Gene Expression Patterns during Long-Term HCV Infection In Vitro

**Figure 3:**
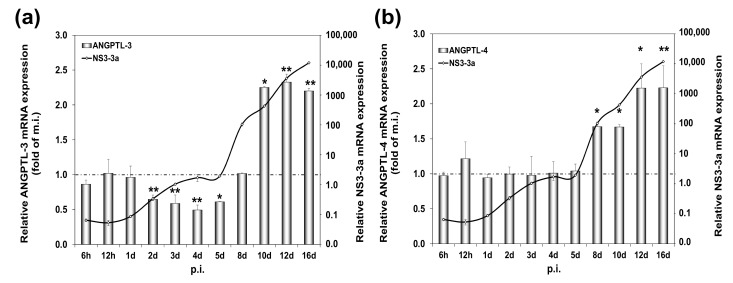
mRNA expression levels of ANGPTL-3 and ANGPTL-4 during long-term HCV infection in vitro. Huh7.5 hepatoma cells infected with HCV-3a were harvested at the stated time points post-infection (p.i.) and subjected to RT-qPCR with (**a**) ANGPTL-3- or (**b**) ANGPTL-4-specific oligonucleotide primers. HCV NS3-3a mRNA expression was used as an indicator of HCV replication (black continuous line on a logarithmic axis). Each bin represents the ratio of the relative mRNA expression in HCV-infected cells to that of the mock-infected control cells (dotted line). Statistically significant differences within groups are denoted by one star (*p*-value ≤ 0.05), or two stars (*p*-value ≤ 0.005).

**Figure A1:**
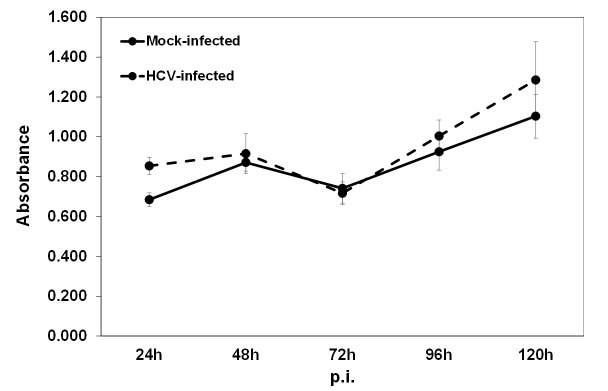
Proliferation assay in early HCV infection. The CellTiter 96^®^ Aqueous One Solution Cell Proliferation Assay (Promega) was used to assess proliferation rates of HCV-infected vs. mock-infected Huh7.5 hepatoma cells at the given time points p.i. The test was carried out according to the manufacturer’s instructions, and the absorbance recorded at 490 nm represents cell growth in the absence (dotted line) or presence (continuous line) of HCV. The experiment was carried out twice in triplicate and did not demonstrate significant differences between the HCV- and the mock-infected cell populations with time.

Having established differential expression patterns of ANGPTL-3 and ANGPTL-4 in HCV infection in vivo, we used Huh7.5 hepatoma cells infected long term (16d) as an in vitro analogy. Past work from our laboratory demonstrated that early HCV infection in vitro (12 h–4 d) with the commonly used HCV JFH-1 (genotype 2a) decreased ANGPTL-3 gene expression [35]. In the present study, we infected the cells with the HCV-3a virus at multiplicity of infection (MOI) = 1. The virus did not affect cell proliferation significantly (Figure A1). As shown in Figure 3a, ANGPTL-3 was down-regulated by at least 50% during the first 5 days, as compared to the mock-infected culture and in agreement with our previous results. To our surprise, after that, ANGPTL-3 mRNA levels rose to a 2.5-fold increase compared to the control from the 8th day until the end of infection on the 16th day (Figure 3a and Supplementary Figure S1a). On the contrary, ANGPTL-4 mRNA expression remained fairly stable during the early stages of HCV infection in vitro but was also up-regulated between the 8th and the 16th day by approximately 1.5- to 2.3-fold (Figure 3b).

It should be noted that Supplementary Figure S1b reconfirms the importance of fasting in the positive regulation of ANGPTL-4 but not ANGPTL-3 hepatic gene expression (Supplementary Figure S1a), as described elsewhere [33]. Taken together, our data suggest that ANGPTL-3 and ANGPTL-4 are differentially expressed during the early stages of HCV infection in vitro, and that the observed differences are genotype-independent for ANGPTL-3. Importantly, the in vitro data do share similarities with the in vivo expression profiles for both ANGPTL proteins.

### 2.4. The Influence of DAA Treatment on ANGPTL-3 and ANGPTL-4 Serum Levels Depends upon the Pre-Treatment Liver Disease Stage

Recently, the discovery of DAAs enabled eradication of the virus in most patients, which in turn led to a reduction in fibrosis levels in patients with milder pre-treatment fibrosis scores [47,48]. Therefore, we investigated the putative effect of DAA administration on the serum concentration of ANGPTL-3 and ANGPTL-4, in clinical samples from various stages of HCV-induced liver disease. A subpopulation of 92 chronic HCV patients out of the original 141, for whom we were able to secure matched blood samples before and after DAA administration, were chosen for this study (see Table 1). All patients included in the study had been treatment-naïve and cleared of the virus. Patients with acute HCV infection and HCV-HCC were not included in this part of the study for statistical and other reasons. Supplementary Table S1 provides information on the demographics and clinical parameters relevant to the DAA study group of 92 participants, showing that no major bias was introduced in the study due to the selection of this subgroup of patients. Table 4 demonstrates the serum levels of both proteins before (BT) and after (AT) the end of treatment. There were statistically significant differences in the median values before and after treatment for all group participants for both ANGPTL-3 (409.7 vs. 377.1 ng/mL, *p*-value < 0.001) and ANGPTL-4 (68.0 vs. 58.3 ng/mL, *p*-value < 0.001), showing that HCV eradication was accompanied by altered ANGPTL-3 and ANGPTL-4 gene expression. However, when we separated measurements according to the fibrosis score, we detected a major difference between the two proteins (Table 4). DAA treatment could not alter ANGPTL-3 levels in patients who received it at the later stages of advanced fibrosis (334.3 vs. 340.6 ng/mL, *p*-value = 0.756) and cirrhosis (418.0 vs. 421.1 ng/mL, *p*-value = 0.121); hence, they possessed a considerably more damaged liver, as opposed to patients with mild liver fibrosis (464.9 vs. 396.3 ng/mL, *p*-value = 0.003). Conversely, ANGPTL-4 levels were significantly changed following DAA administration in clinical samples from all stages of fibrosis, i.e., mild fibrosis (63.4 vs. 54.8 ng/mL, *p*-value = 0.031), advanced fibrosis (92.9 vs. 75.5 ng/mL, *p*-value = 0.003) and cirrhosis (94.6 vs. 60.7 ng/mL, *p*-value = 0.014). Table A1 in Appendix A.2 describes a relationship between the number of patients who had altered ANGPTL-3 and ANGPTL-4 levels and fibrosis stage following DAA administration.

### 2.5. Establishment of an In Vitro HCV Clearance Model

**Figure 4:**
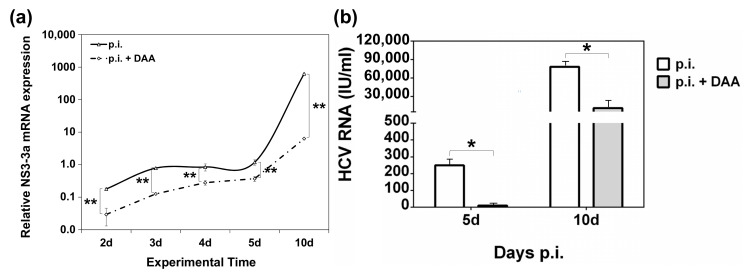
(**a**) HCV-3a replication levels before and after HCV eradication in an in vitro HCV clearance model. Huh7.5 hepatoma cells were infected with HCV-3a and treated with a mixture of HCV antiviral compounds (DAA) or left untreated. The infected cells were harvested at the stated time points p.i. and subjected to RT-qPCR with NS3-3a-specific oligonucleotide primers as an indicator of HCV replication in the presence (continuous line) and absence (dotted line) of the antiviral treatment. Relative mRNA expression for the HCV NS3-3a gene is plotted on a logarithmic axis. Statistically significant differences between the HCV NS3-3a mRNA values from DAA-treated and untreated infected cells are denoted by one star (*p*-value ≤ 0.05), or two stars (*p*-value ≤ 0.005). (**b**) HCV infectivity following DAA administration in an in vitro HCV clearance model. Supernatants from the HCV-3a-infected hepatoma cell cultures described above were collected before (white bars) and after (grey bars) DAA treatment and used for the isolation of viral RNA. Statistically significant differences within groups are denoted by one star (*p*-value ≤ 0.05), or two stars (*p*-value ≤ 0.005).

The role of DAA-mediated eradication of HCV in the gene expression profile of ANGPTL-3 and ANGPTL-4 was investigated in vitro. For this, we created an in vitro HCV clearance model, by treating HCV-infected Huh7.5 hepatoma cells with the commercially available pangenotypic inhibitory compounds glecaprevir and pibrentasvir. Cytotoxicity analysis revealed no significant damage to Huh7.5 cells with the administered concentration of 3.5 µM, since the drug-induced cell death was comparable to that of the vehicle dimethyl sulfoxide DMSO (Supplementary Figure S2). Then, the cells were infected with HCV-3a, treated with 3.5 µM of the inhibitor or (DMSO) and harvested at specific time points between 2 and 10 days. Figure 4a shows HCV replication, as measured by HCV NS3-3a mRNA levels before and after DAA treatment. While the vehicle did not affect NS3-3a mRNA levels (data not shown), a dramatic decrease in HCV replication was observed at every time point of infection, spanning from 70% to 99% in the presence of the drug. Furthermore, HCV RNA measurements in cell culture supernatants at 5 days and 10 days before and after DAA treatment with a commercially available diagnostic kit confirmed that the reduced viral replication corresponded to diminished HCV infectivity, ranging between 86% and 96%, as expected (Figure 4b).

### 2.6. Modelling the Effect of DAA Treatment on ANGPTL-3 and ANGPTL-4 Gene Expression of HCV-Infected Hepatocytes In Vitro

**Figure 5:**
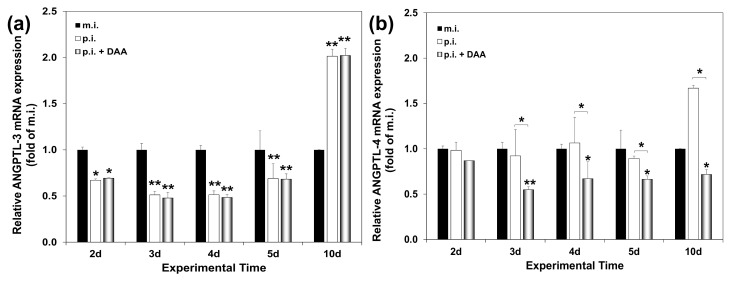
mRNA expression levels of ANGPTL-3 and ANGPTL-4 before and after HCV eradication in an in vitro HCV clearance model. Huh7.5 hepatoma cells infected with HCV-3a were harvested at the stated time points p.i. and subjected to RT-qPCR with (**a**) ANGPTL-3- or (**b**) ANGPTL-4-specific oligonucleotide primers. White and grey bins represent the ratio of relative mRNA expression in HCV-infected (p.i.) or HCV-cured (p.i. + DAA) cells to that of the mock-infected (m.i.) control (black bins). Statistically significant differences within groups are denoted by one star (*p*-value ≤ 0.05), or two stars (*p*-value ≤ 0.005).

Having validated our in vitro HCV clearance model, we proceeded to determine ANGPTL-3 and ANGPTL-4 mRNA levels in HCV-3a-infected hepatocytes before and after DAA administration by RT-qPCR. Figure 5a demonstrates that compared to the mock-infected controls (denoted by black bars), ANGPTL-3 mRNA expression was found to be down-regulated to the same degree in both infected (white bars) and cured (black bars) cells, at all the inspected time points. On the other hand, DAA treatment of the infected cells (grey bars) reduced ANGPTL-4 mRNA by 30–50% (between the 3rd and 10th days p.i.) compared to the expression of the mock-infected (black bars) and the HCV-infected cells (white bars) (Figure 5b). Notably, incubation of Huh7.5 cells with the DAA inhibitor did not alter ANGPTL-3 and ANGPTL-4 gene expression at any time point examined (Supplementary Figure S3). Overall, we conclude that despite the diminished viral replication and infectivity achieved in our in vitro HCV clearance model, the alterations in ANGPTL-3 gene expression observed during HCV infection persisted, in resemblance to the data collected from HCV patients. Importantly, the differential effect of DAA treatment on ANGPTL-3 and ANGPTL-4 expression was also evident in vitro.

### 2.7. Does Serum Expression of ANGPTLs during HCV Infection Correlate with Specific Demographic, Clinical and Viral Parameters?

The univariate analyses presented in Table 5 comprise the calculation of the relevant correlation coefficients and *p*-values that describe the relationship of ANGPTL-3 and ANGPTL-4 serum concentrations before and after antiviral treatment with HCV VRL, the genotype, gender and age of the patients and the hepatic fibrosis stage. ANGPTL-3 levels were positively correlated with age both before and after DAA treatment, while the post-treatment concentration was weakly associated with gender. For ANGPTL-4, we observed a weak negative correlation between ANGPTL-4 post-treatment and patient gender. Importantly, there were significant associations between ANGPTL-3 and ANGPTL-4 concentrations before treatment and fibrosis stage. Interestingly, after treatment, this association with fibrosis was preserved only for ANGPTL-4 but became much weaker. We were unable to detect any statistically significant correlations with viral parameters with this type of analysis.

In order to understand which of the factors used in the univariate correlations could be associated with ANGPTL-3 and ANGPTL-4 levels before and after DAA treatment while adjusting for all the others, we proceeded to carry out multivariate analysis by developing linear regression models. The BT concentrations were also introduced as an independent variable in the models computing the AT predictors. As shown in Table 6, ANGPTL-3 levels before DAA treatment were positively correlated with age. Importantly, advanced fibrosis reduced ANGPTL-3 levels by 128.4 units (95% C.I. = −216.7 to −40.2, *p*-value = 0.005), as compared to mild fibrosis. Such a reduction was not found statistically significant for the cirrhotic patients. The ANGPTL-3 serum concentrations after treatment correlated positively with the HCV VRL measured before viral eradication, since patients with a high VRL tended to have higher ANGPTL-3 levels than patients with a low VRL after treatment. Finally, the ANGPTL-3 levels before treatment seemed to offer a small positive but significant input to defining the post-treatment serum concentration of the protein.

As far as ANGPTL-4 BT levels are concerned, we also detected positive correlations with fibrosis stage. In fact, patients with advanced fibrosis and cirrhosis would increase their ANGPTL-4 serum concentration by approximately 31 units, in comparison with the mildly fibrotic patients (95% C.I. = 8.2 to 54.9, *p*-value = 0.009 for advanced fibrosis, and 95% C.I. = 10.3 to 53.0, *p*-value = 0.004 for cirrhosis). ANGPTL-4 levels post-treatment were found to be positively correlated with age and BT levels. Interestingly, female HCV patients tended to have a lower ANGPTL-4 serum concentration after treatment as compared to male patients. Furthermore, the high HCV VRL before viral eradication decreased the AT levels of ANGPTL-4, in contrast with patients with a low VRL. The HCV genotype was not correlated with ANGPTL levels either before or after treatment. Notably, there were no multicollinearity issues with our models, since all variance inflation factors (VIF) values for all entered variables were below 2.0. Taken together, the predictors used in the models could explain about 24% of the observed ANGPTL-3 levels before DAA treatment and 44.1% after treatment. The corresponding values for ANGPTL-4 were 11.3% and 60.6% pre- and post-treatment, respectively.

### 2.8. Differential Association of ANGPTL-3 and ANGPTL-4 Pre-Treatment Levels with the Profibrotic Cytokine TGF-β

**Figure 6:**
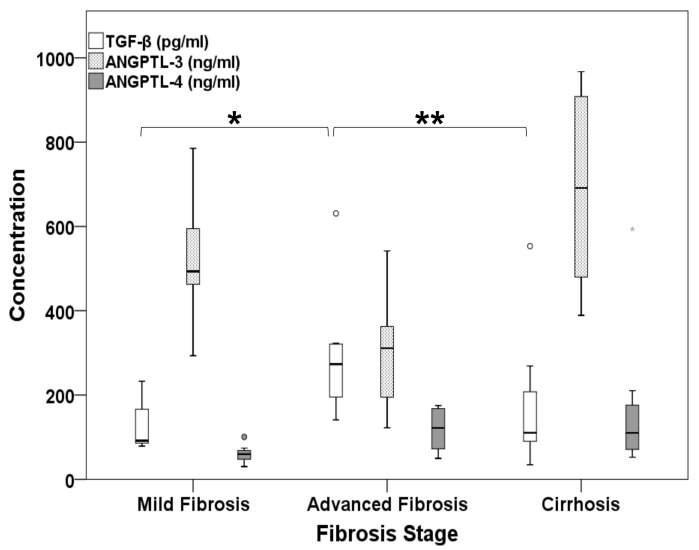
Serum levels of the key profibrotic cytokine TGF-β in correlation with ANGPTL-3 and ANGPTL-4 in HCV patients with mild fibrosis, advanced fibrosis and cirrhosis. The naturally active form of TGF-β (white box plots) was determined by sandwich ELISA from the sera of HCV patients prior to DAA treatment and is plotted together with ANGPTL-3 (light grey box plots) and ANGPTL-4 (dark grey box plots) levels against fibrosis stage. Statistically significant differences within groups are denoted by one star (*p*-value ≤ 0.05), or two stars (*p*-value ≤ 0.005).

TGF-β mediates the activation and differentiation of HSCs to myofibroblasts and the onset of liver fibrosis, together with a vast repertoire of other cellular and immune responses [49,50]. We probed the relationship between ANGPTLs and fibrosis further by correlating TGF-β expression with the levels of ANGPTL-3 and ANGPTL-4 using sera from the group of patients we conducted the DAA study on. Similar to ANGPTLs, we used a DuoSet^®^ sandwich ELISA development kit (R&D Systems, Minneapolis, MN, USA) to determine the concentration of active TGF-β in patient sera. Figure 6 portrays the TGF-β sera concentrations in untreated HCV patients with mild (F0–F2)/advanced (F3) fibrosis and cirrhosis (F4) (white box plots), together with the respective ANGPTL-3 (light grey box plots) and ANGPTL-4 (dark grey box plots) levels for comparative purposes. A Kruskal–Wallis test for independent samples (*p*-value = 0.011) followed by post hoc pairwise comparisons revealed that TGF-β was significantly more elevated in advanced fibrosis than in mild fibrosis and cirrhosis (273.3 vs. 91.7 pg/mL, *p*-value = 0.004, and 273.3 vs. 110.4 pg/mL, *p*-value = 0.028, respectively). Notably, TGF-β was found to be negatively correlated with ANGPTL-3 (r_s_ = −0.482, *p*-value = 0.027) and positively correlated with ANGPTL-4 BT levels (r_s_ = 0.486, *p*-value = 0.016), as depicted in Figure 6. These results are in agreement with the multiple regression analysis data and hint towards a strong but dissimilar interrelation between ANGPTLs and the HCV-mediated development of hepatic fibrosis. We could not find any correlation between the serum levels of TGF-β and the ANGPTLs in any disease stage after the completion of DAA treatment (r_s_ = −0.194, *p*-value = 0.376 for the TGF-β/ANGPTL-3 pair, and r_s_ = 0.097, *p*-value = 0.667 for the TGF-β/ANGPTL-4 pair).

## 3. Discussion

Manipulation of key lipid metabolism regulatory genes has been strategically pursued by HCV for the creation of lipid-rich cellular surroundings needed for the successful completion of all steps of the viral life cycle [51,52]. Host lipid metabolism reprogramming is deemed important for the advancement of HCV-induced liver disease and the initiation of hepatocarcinogenesis not only in cirrhotic patients with active infection [16,17] but also after DAA-mediated eradication of the virus in genetically susceptible individuals [53]. The present study mainly determined the serum levels of the host lipid regulators ANGPTL-3 and ANGPTL-4 in HCV patients throughout the natural history of hepatitis C, from acute infection to HCC. The effect of HCV on ANGPTL-3 and ANGPTL-4 gene expression during long-term infection of hepatoma cells in vitro was also studied.

Currently, non-invasive Fibroscan^®^ transient elastography accurately differentiates between a healthy liver, severe fibrosis and cirrhosis [54], thereby assisting with the management of liver damage and HCC prevention [55]. Our sera samples were stratified for hepatic disease progression by using the METAVIR fibrosis score (F0–F4) [56], after Fibroscan^®^ measurements in treatment-naïve patients. Chronic HCV patients were grouped further into the three clinically relevant categories of mild fibrosis (F0, F1 and F2), advanced fibrosis (F3) and cirrhosis (F4). The F3 stage is known as the “bridging” step between complication-free liver disease and cirrhosis, the latter characterised by a deformed liver architecture, increased chances for HCC and severe systemic pathologies [57,58]. We chose to differentiate F3 patients from the other stages because although F3 patients tend to have better clinical outcomes and overall survival [57], many clinical researchers recommend that they should also be monitored for HCC development post-cure [19,59]. Additionally, there is still not enough information available about established HCV-mediated molecular mechanisms of tumourigenesis at this stage. Our findings show that ANGPTL-3 was the least expressed in F3 patients compared to all other stages. Conversely, ANGPTL-4 displayed an increasing tendency from the early (acute infection/mild fibrosis) to late (cirrhosis/HCC) stages, but in a non-linear way since it peaked in patients with advanced fibrosis. Interestingly, both ANGPTL-3 and ANGPTL-4 serum levels did not statistically differ between the early stages of acute infection and mild fibrosis or the late stages cirrhosis and HCC (Figure 2). Thus, the F3 stage represents an important threshold for ANGPTL serum expression and accentuates the differential regulation of ANGPTLs in HCV infection.

To our knowledge, one other study has reported ANGPTL-3 and ANGPTL-4 expression in HCV patients. However, the authors did not take into account the input of fibrosis and reported an increased expression of both ANGPTLs in chronic hepatitis C infection and HCV-induced HCC compared to healthy blood donors [60]. Given the mounting evidence for the role of ANGPTL-3 and ANGPTL-4 in cancer [36,40], perhaps the hepatic microenvironment shaped by advanced fibrosis in F3 HCV patients could be the one that dictates the depth of ANGPTLs’ involvement in HCC development. Importantly, the differential expression of ANGPTLs throughout the natural history of HCV infection could reflect direct regulation by both HCV and the infected hepatocyte. This could be especially true for ANGPTL-4 that has been shown to be modulated by inflammation, hypoxia, cytokines and other cellular processes [39,41] and warrants further investigation in itself.

At the same time, we determined ANGPTL-3 and ANGPTL-4 gene expression in mRNA samples isolated from sixteen day-long infections of permissive hepatoma cells with HCV-3a. Compared to mock-infected cells, ANGPTL-3 was down-regulated almost by half in early infection, while ANGPTL-4 remained unchanged (Figure 3). Hence, the observed decrease in ANGPTL-3 expression may be important for the establishment of the HCV life cycle, due to ANGPTL-3′s role as an inhibitor of lipid clearance by the liver [61]. On the other hand, ANGPTL-4 was not decreased by HCV, despite functional similarities with ANGPTL-3 in lipid regulation. We cannot rule out the possibility that HCV alters ANGPTL-4 gene expression in macrophages, which are also infected and used as reservoirs by the virus [62]. Notably, both genes showed a delayed increase in late HCV infection in vitro, and this could be related to the different viral needs during persistent infection that favour inducers rather that inhibitors of these proteins in the liver. In any case, the ANGPTL-3 and ANGPTL-4 expression patterns in vitro bear similarities with the corresponding ones in vivo.

Next, serum levels of ANGPTL-3 and ANGPTL-4 were measured in our HCV patients at the end of a 12-week treatment period with DAAs. Compared to pre-treatment levels, ANGPTL-4 was mostly altered independently of the fibrosis stage. Surprisingly, this did not happen to ANGPTL-3 when treatment was administered to patients with advanced fibrosis and cirrhosis. Evidently, retention of ANGPTL-3 baseline values after treatment was linked to more severe disease. In contrast, ANGPTL-4 expression was subjected to a “corrective” adjustment. Furthermore, DAA administration in vitro did not revert the HCV-mediated regulation of ANGPTL-3 gene expression towards that displayed by mock-infected cells. Conversely, ANGPTL-4 was found reduced, for the first time, upon HCV elimination (Figure 5). Our data suggest that ANGPTL-3 may be one of many genes belonging to the host transcriptome, secretome, epigenome and immunome that retain their expression levels after successful HCV eradication in patients with advanced liver damage. These genes are believed to be part of a residual HCV fingerprint that has been suggested to promote hepatocarcinogenesis in some cured patients [21,22,63,64]. Surprisingly, similar persistently expressed immunoregulatory signatures were even discovered in acutely infected HCV patients who received early DAA treatment [65].

Many researchers have reported recovery from HCV-induced hypolipidaemia after DAA treatment [66,67,68,69,70]. However, most studies showed that ANGPTL-targeted lipid species, such as triglycerides and VLDL cholesterol, remained the same before and after DAA treatment, even at 24-week follow-ups [66,67,68,71]. Alternatively, a moderate post-cure increase was noted only in patients with mild fibrosis [70]. This partial recovery of circulating lipids is accompanied by increasing steatosis in patients with or without co-existing predisposing factors, through unclear mechanisms [72,73]. Although we did not determine the concentration of lipids and cholesterol in our samples, the HCV-dictated ANGPTL-3 levels remaining after viral eradication could be causally involved, at least in part, in shaping specific lipid species and steatosis after treatment. In the long run, ANGPTL-3 levels after HCV eradication could promote lipotoxicity-related hepatocarcinogenesis, thereby strengthening ANGPTL-3′s emerging role in cancer [36]. On the contrary, ANGPTL-4′s expression profile after DAA treatment indicated that it probably does not belong to this HCV-preserved pool of genes, and that the observed decrease in vivo and in vitro could be dictated by other factors in the host environment, such as residual inflammatory activity recorded in the serum of DAA-treated patients [74,75].

Correlation and regression analyses (see Table 5 and Table 6) showed that patient gender and age may be associated, to some extent, with ANGPTL-3 and ANGPTL-4 levels during HCV infection or after HCV eradication. Such relationships have been described in studies concerning healthy populations [76,77]. They may be due to the higher activity of target lipases LPL and HL seen in women or reflect inherent changes in lipid metabolism occurring with age [78]. Furthermore, high VRL was a significant predictor for ANGPTL-3 and ANGPTL-4 levels after treatment, but not during infection, as would have been expected. It remains controversial whether the HCV VRL is independently associated with disease progression and HCC development [79,80]. However, it could somehow shape the residual fingerprint left by the virus, through epigenetic modifications, exosomal cargo manipulation and mechanisms that have not been delineated yet.

Finally, another major finding of this study was that ANGPTL-3 and ANGPTL-4 expression was associated with fibrosis during HCV infection. TGF-β is considered to be the master regulator of fibrosis. It has been strongly implicated in the reduction of inflammation, promotion of fibrinogenesis, liver cancer development and facilitation of metastasis depending on the cellular milieu [58,81]. Its involvement in liver fibrosis and HCV-mediated disease progression has also been verified [82,83,84]. Structural activation of TGF-β is strictly regulated and directly dictates its cellular functions [49]. Therefore, we determined the levels of the active TGF-β peptide in patients with mild/advanced fibrosis and cirrhosis in an effort to see whether TGF-β is the link between fibrosis and the expression patterns of ANGPTL-3 and ANGPTL-4. Our results show that TGF-β was significantly elevated in the serum of F3 patients compared with the other fibrosis stages (Figure 6). This agrees with past studies demonstrating that TGF-β may be overexpressed in regenerating liver nodules amply found in advanced fibrosis as promoters of wound healing [81,85]. ANGPTLs were inversely associated with TGF-β levels before treatment. In fact, ANGPTL-3 was negatively correlated with TGF-β, and ANGPTL-4 displayed a positive association with the factor (Figure 6). In addition, we did not detect any correlation between the post-DAA concentrations of the examined proteins. ANGPTL-4 has already been shown to be up-regulated by TGF-β in breast and bone cancer [86,87], but the relationship between ANGPTL-3 and TGF-β is not so clear. Unpublished observations from our laboratory suggested a profound TGF-β-mediated decrease in ANGPTL-3 mRNA in hepatoma cells [88]. Therefore, the TGF-β-mediated regulation of ANGPTL-3 and -4 gene expression may imply a causal relationship between these molecules and the cytokine. It could also partially explain the correlation between ANGPTLs and fibrosis throughout the natural history of HCV infection, but it needs to be verified in every individual fibrosis stage of the disease. Notably, the receding effect of fibrosis on ANGPTL-4 expression post-cure observed in univariate analysis could become insignificant in patients who achieve fibrosis regression with time.

## 4. Materials and Methods

### 4.1. Clinical Samples and Other Materials

A total of 141 HCV patients at various stages of infection were included in this study. All of them were HIV- and HBV-negative. The patients had their blood drawn in the morning on an empty stomach by medical staff of the 2nd Department of Internal Medicine, Hippokration Hospital, Medical School of Athens. Aliquots of sera samples prepared from whole blood were kept at −80 °C and thawed only once for utilisation in ELISA experiments. Their use in this study was approved by the Bioethics Committee of the Hellenic Pasteur Institute, taking into account the written advice of the Ethics Committee of Hippokration Hospital of Athens. A written informed consent was signed by all patients in accordance with the Declaration of Helsinki.

The HCV DBN3a (genotype 3a, HCV-3a) infectious clone was generously provided by Prof. J. Bukh (Department of Immunology and Microbiology, University of Copenhagen, Denmark). Unless otherwise stated, all other materials were from Thermo Fisher Scientific (Waltham, MA, USA).

### 4.2. Cell Lines and Viral Infections

The Huh7.5 hepatoma cells were maintained in high-glucose Dulbecco’s Modified Eagle Medium (DMEM) supplemented with 10% (*v*/*v*) heat-inactivated foetal bovine serum, 2 mM glutamine, 100 U/mL penicillin/streptomycin and non-essential amino acids.

Preparations of viral stocks for the HCV-3a virus were prepared according to the previously published protocol of Kato and colleagues [89]. Viral infections were carried out in the permissive Huh7.5 hepatoma cell line, using 800,000 cells/well and MOI = 1 in 6-well plates. After 4 h, the viral particles were removed, and fresh culture medium was added. For long-term infections, both HCV- and mock-infected cells were subcultured every 4–5 days. Cells were harvested at various time points ranging between 6 h and 16 d p.i.

### 4.3. In Vitro HCV Clearance Model

For the establishment of an in vitro HCV clearance model, HCV-infected Huh7.5 hepatoma cells were treated with a commercially available combination of the direct-acting antiviral compounds glecaprevir (100 mg) and pibrentasvir (40 mg), which are pangenotypic pharmacological inhibitors of HCV NS3/4A and HCV NS5A proteins, respectively. Thus, a single tablet was dissolved in DMSO to produce a working stock solution of 1.2 mM, which was further diluted in cell culturing medium to provide a clinically relevant concentration of 3.5 µM [90]. The CytoTox 96 Non-Radioactive Cytotoxicity Assay (Promega, Fitchburg, WI, USA) was used to ensure that this concentration would not confer any cytotoxic effects to the cells during their incubation with the drug. Briefly, 3.5 µM DAA or its equivalent volume in vehicle (DMSO) was added in 10,000 cells/well in a 96-well plate. Upon cell lysis at 24 h, 48 h, 72 h and 96 h post-drug addition, any cytosolic lactate dehydrogenase (LDH) released in culture supernatants was measured by a coupled enzymatic assay that resulted in the conversion of a tetrazolium salt substrate into a red formazan compound. The reaction was stopped by the addition of 1M acetic acid, and the absorbance measured at 490 nm was proportional to the number of lysed cells.

Subsequently, Huh7.5 cells were infected as previously described with HCV-3a for the indicated time points. A single dose of 3.5 µM DAA or vehicle (DMSO) was added to the cultures 12 h before cell harvesting. Mock-infected cultures were treated similarly.

### 4.4. mRNA Analysis

Total RNA was isolated from cells using NucleoZOL (Macherey-Nagel, Düren, Germany) according to the manufacturer’s instructions. Reverse transcription reactions were carried out using 1 µg total RNA, p(dN)_6_ random hexamers (Roche, Basel, Switzerland) and Moloney murine leukaemia virus (MMLV) reverse transcriptase (Promega, Fitchburg, WI, USA). The prepared cDNA was used in qPCR amplification reactions with the Kapa^®^ SYBR Fast Master Mix (Kapa Biosystems, Wilmington, MA, USA) in a Corbett Rotor Gene 6000 thermocycler (Qiagen, Hilden, Germany). The host gene-specific primers used were ANGPTL-3F: 5′ CCA GAA CAC CCA GAA GTA ACT 3′, ANGPTL-3R: 5′ TCT GTG GGT TCT TGA ATA CTA GTC 3′; ANGPTL-4F: 5′ GCA GGA TCC AGC AAC TCT TC 3′, ANGPTL-4R: 5′ AAA CTG GCT TTG CAG ATG CT 3′ [91]; and 18S rRNAF: 5′ CTC AAC ACG GGA AAC CTC AC 3′, 18S rRNAR: 5′ CGC TCC ACC AAC TAA GAA CG 3′ [92]. The viral NS3 gene utilised to monitor HCV infection offered a measure of viral replication and was amplified with the oligonucleotide primer pair NS3-3aF: 5′ GCA GCG GTA AGA GCA CAA AG 3′, NS3-3aR: 5′ TAG GCA CGC GAC ATG AAA GA 3′ for HCV-3a. Results were analysed with the internal standard curve method and normalised to the 18S rRNA house-keeping gene to provide relative mRNA expression. All experiments were carried out three times in triplicate.

### 4.5. ELISA Analysis

ANGPTL-3, ANGPTL-4 and active TGF-β serum concentrations were measured with the DuoSet^®^ DY3829, DY3485 and DY240 sandwich ELISA development kits (R&D Systems, Minneapolis, MN, USA), respectively, using the original sandwich ELISA protocols recommended by the supplier with modifications. Sera samples were thawed only once on ice and centrifuged at 10,000× *g* for 5 min at 4 °C to remove any debris. Lipaemic and haemolysed samples were excluded. Matched (before and after treatment) sera samples were always run together on the same plate. Briefly, capture antibody at a concentration of 4 µg/mL in phosphate buffer saline (PBS) for ANGPTL-3, 1.4 µg/mL for ANGPTL-4 and 2.5 µg/mL for TGF-β was used to coat 96-well polystyrene high-binding plates overnight at 4 °C or r.t. The plates were washed twice with washing buffer (0.1% (*v*/*v*) PBS-Tween-20; pH 7.2), and non-specific binding sites were blocked with 1% (*w*/*v*) bovine serum albumin (BSA) in PBS (ANGPTLs) or 5% (*v*/*v*) Tween-20 in PBS (TGF-β) for 2 h at r.t. Seven- or eight-point standard curves were used for the determination of ANGPTL-3 (31.25–2000 pg/mL), ANGPTL-4 (0.625–80 ng/mL) and TGF-β (7.8–1000 pg/mL) serum concentrations. The serum dilution factors used were 200- (ANGPTL-3) and 10-fold (ANGPTL-4) in reagent diluent (0.5% (*w*/*v*) BSA, 0.1% (*v*/*v*) Tween-20 in PBS). For TGF-β, we used a 10-fold serum dilution in an appropriate reagent diluent provided by the supplier. Standards and serum samples were transferred simultaneously on ELISA plates and incubated overnight at 4 °C (ANGPTLs) or 2 h at r.t. (TGF-β) under gentle agitation. Next, the plates were washed four times with washing buffer, avidin-conjugated detection antibody at a concentration of 400 ng/mL for ANGPTL-3, 200 ng/mL for ANGPTL-4 and 50 ng/mL for TGF-β in reagent diluent was applied and the plates were incubated for a further 2 h at r.t. The plates were washed twice with washing buffer, and streptavidine-conjugated horseradish peroxidase solution (1:200 in reagent diluent for ANGPTLs, and 1:40 for TGF-β) was added for 20–30 min at r.t. For the detection of peroxidase activity, the plates were washed twice with washing buffer and once with PBS, and 3,3′,5,5′-tetramethylbenzidine (TMB) substrate reagent was added for 15–20 min. The reaction was stopped with 1M H_2_SO_4_, and the absorbance was measured at 450 nm on a microplate reader (Bio-Rad, Hercules, CA, USA). All measurements were carried out in duplicate and averaged. Serum concentrations were calculated using a 5-parameter logistic regression slope curve.

### 4.6. Viral Infectivity Measurements

Viral RNA from cell culture supernatants was isolated using the QIAamp Viral RNA kit (Qiagen, Hilden Germany), according to the manufacturer’s instructions. Viral infectivity was estimated with quantitation of the HCV RNA in infected and cured samples with the Artus^®^ HCV RG RT-PCR kit (Qiagen, Hilden, Germany).

### 4.7. Statistical Analysis

Datasets were tested for normality with the Shapiro–Wilk test. Normally distributed parameters were described by mean and SD, data that did not fit the normal distribution were presented by their median and their first–third quartiles and categorical data were summarised by percentages. For categorical variables, we used the χ^2^ goodness of fit test to assess observed vs. expected frequencies across groups. The Kruskal–Wallis test for independent samples was used for comparison between three or more groups followed by post hoc pairwise comparisons with Dunn’s test. In the case of paired samples collected from patients before and after treatment with DAAs or comparisons of means between two groups of normally distributed data from in vitro experiments, we used a related samples Wilcoxon signed rank test or Student’s *t*-test, respectively. Univariate correlation analyses between variables were carried out using Spearman’s rank correlation or point biserial or eta coefficients, depending on the nature of the variables examined. Finally, we carried out multivariate linear regression in order to assess the dependence of the ANGPTL-3 and ANGPTL-4 serum concentration on various clinical and viral parameters. For categorical predictors with multiple levels, we set one level as a reference group for the others. Multicollinearity issues of the model were assessed by calculation of the respective VIF. In all cases, statistical significance is denoted as a *p*-value ≤ 0.05 and reported by * if *p*-value ≤ 0.05 and ** if *p*-value ≤ 0.005 in figure graphs.

## 5. Conclusions

In conclusion, we investigated, for the first time, ANGPTL-3 and ANGPTL-4 expression throughout the natural history of hepatitis C in vivo and long-term HCV infection in vitro and then determined the corresponding ANGPTL levels after treatment with DAAs. The observed HCV-mediated differential regulation of these genes depended on the fibrosis stage, with advanced fibrosis proven to be pivotal in ANGPTL-3 and -4 expression’s fate. Importantly, ANGPTL-3 levels were not subjected to post-cure corrective adjustment in patients with advanced liver disease and after HCV clearance in vitro. Thus, ANGPTL-3 may be part of the residual HCV molecular fingerprint that could contribute to persistent deregulation of lipid metabolism in cured individuals and predispose some of them to HCC development.

## Figures and Tables

**Table 1:** Total demographics of the study.

Clinical Parameter	Mean ± SD	*p*-Value
Age (years)	49 ± 13	n/a
ALT (IU/mL)	95 ± 78	n/a
**Clinical Characteristic**	***n* = 141 (%)**	
Male/Female	69 (48.2%)/72 (51.1%)	0.801
DAA treatment BT–AT ^§^ matched pairs	92 (65.2%)	n/a
VRL (BT)—low (<1 × 10^6^ IU/mL)	56 (39.7%)	0.157
VRL (BT)—high (≥1 × 10^6^ IU/mL)	72 (51.1%)	
VRL (BT)—Not determined	13 (9.2%)	
Genotype—HCV 1a	25 (17.7%)	0.057
Genotype—HCV 1b	33 (23.4%)	
Genotype—HCV 2a	15 (10.6%)	
Genotype—HCV 3	35 (24.8%)	
Genotype—HCV 4	27 (19.2%)	
Genotype—Not determined	6 (4.3%)	
Disease Stage (Fibrosis Score *)		
Acute Infection (F0–F1)	6 (4.2%)	0.000
Mild Fibrosis (F0/F1/F2)	63 (44.7%) (12/24/27)	
Advanced Fibrosis (F3)	17 (12.1%)	
Cirrhosis (F4)	42 (29.8%)	
HCC (F4)	13 (9.2%)	

n/a: not applicable, SD: standard deviation, ALT: alanine aminotransferase, ^§^ BT–AT: before treatment–after treatment, VRL: viral load, * fibrosis score key: F0: 2–4, F1: 4–7, F2: 7–9, F3: 9–12, F4 > 12.

**Table 2:** Study demographics by disease stage.

Clinical Parameter	Acute	Mild Fibrosis	Advanced Fibrosis	Cirrhosis	HCC	*p*-Value
	**Mean ± SD**					
Age	34.7 ± 8.5	49.7 ± 11.0	51 ± 9.5	56.1 ± 15.5	65.2 ± 8.9	0
ALT (IU/mL)	725.4 ± 300.1	57.1 ± 43.2	73.1 ± 69.0	90.7 ± 85.1	63.8 ± 47.8	0
**Clinical**	*n* * (%)					
**Characteristic**						
Gender (Male)	6 (100%)	23 (36.5%)	9 (52.9%)	21 (50%)	10 (76.9%)	0.006
VRL-low (<1 × 10^6^ IU/mL)	2 (33.3%)	28 (44.4%)	6 (35.3%)	18 (42.8%)	2 (15.4%)	-
VRL-high (≥1 × 10^6^ IU/mL)	2 (33.3%)	33 (52.4%)	11 64.7%)	23 (54.8%)	3 (23.1%)	0.951
Not determined	2 (33.4%)	2 (3.2%)	n/a	1 (2.4%)	8 (61.5%)	
Genotype						
HCV1a	-	12 (19.1%)	4 (23.5%)	9 (21.4%)	-	0.347
HCV1b	2 (33.3%)	13 (20.6%)	4 (23.5%)	13 (31.0%)	1 (7.6%)	
HCV2a	-	11 (17.5%)	1 (5.9%)	3 (7.1%)	-	
HCV3	4 (66.7%)	14 (22.2%)	4 (23.5%)	10 (23.8%)	3 (23.1%)	
HCV4	-	13 (20.6%)	4 (23.5%)	7 (16.7%)	3 (23.1%)	
Not determined	-	-	-	-	6 (46.2%)	

* *n*: number of participants per disease stage as presented in Table 1.

**Table 3:** ANGPTL-3 and ANGPTL-4 serum levels during the course of natural HCV infection.

Disease Stage	Acute Infection	Mild Fibrosis	Advanced Fibrosis	Cirrhosis	HCC	*p*-Value *
	**Median (IQR) in ng/mL**					
ANGPTL-3 BT ^1^	563.6 (185.1–682.8)	493.3 (361.5–82.8)	332.1 (228.3–394.4)	455.3 (347.1–575.9)	375.8 (289.3–450.3)	0.002
ANGPTL-4 BT ^1^	60.1 (24.9–89.0)	60.1 (40.6–74.1)	102.8 (65.9–129.5)	78.5 (53.1–113.1)	86.0 (49.7–133.2)	0.002

IQR: interquartile range, ^1^ BT: all measurements were carried out in patients who had not previously received any treatment. * Between groups.

**Table 4:** ANGPTL-3 and ANGPTL-4 levels at various stages of HCV infection before and after DAA administration.

Disease Stage	ANGPTL-3			ANGPTL-4		
	Median (IQR) in ng/mL					
	BT ^1^	AT ^2^	*p*-Value *	BT ^1^	AT ^2^	*p*-Value *
All	409.7 (346.4–548.6)	377.1 (300.6–483.7)	0.000	68.0 (53.6–102.5)	58.3 (46.4–81.6)	0.000
Mild Fibrosis	464.9 (346.4–548.6)	396.3 (300.3–483.7)	0.003	63.4 (47.8–75.4)	54.8 (40.2–68.2)	0.031
Advanced Fibrosis	334.3 (225.0–397.9)	340.6 (249.5–365.7)	0.756	92.9 (64.9–134.6)	75.5 (51.0–102.0)	0.003
Cirrhosis	418.0 (322.2–549.5)	421.1 (322.0–509.0)	0.121	94.6 (56.7–139.8)	60.7 (48.6–81.6)	0.014

^1^ BT: patients who had not previously received any treatment, ^2^ AT: the same patients following DAA treatment. * Between BT and AT groups.

**Table 5:** Univariate association analysis of clinical and infection parameters with ANGPTL-3 and ANGPTL-4 sera levels in matched samples of chronic HCC patients before (BT) and after (AT) treatment with DAAs.

Parameter	ANGPTL-3				ANGPTL-4			
	BT		AT		BT		AT	
	Coefficient	*p*-Value	Coefficient	*p*-Value	Coefficient	*p*-Value	Coefficient	*p*-Value
VRL H–L *	−0.037 ^2^	0.737	0.061 ^2^	0.578	0.124 ^2^	0.254	0.024 ^2^	0.829
Genotype	0.129 ^3^	0.844	0.070 ^3^	0.982	0.248 ^3^	0.249	0.269 ^3^	0.172
Gender	0.173 ^2^	0.108	**0.224** ^2^	0.036	−0.130 ^2^	0.227	**−0.210** ^2^	0.050
Age	**0.375** ^1^	0.000	**0.427** ^1^	0.000	0.091 ^1^	0.407	−0.031 ^1^	0.771
Fibrosis	**0.320** ^3^	0.010	0.192 ^3^	0.320	**0.414** ^3^	0.000	**0.268** ^3^	0.042

Coefficient key: ^1^ Spearman’s rank correlation coefficient r_s_, ^2^ point biserial coefficient r_pb_, ^3^ eta coefficient (η). Statistically significant associations are presented in bold. *: viral load high–low.

**Table 6:** Identification of independent parameters that may influence ANGPTL-3 and ANGPTL-4 serum levels before and after viral eradication by multiple linear regression analysis.

Parameters	ANGPTL-3						ANGPTL-4					
	BT (R^2^ = 0.243)			AT (R^2^ = 0.441)			BT (R^2^ = 0.113)			AT (R^2^ = 0.606)		
	B	*p*-Value	95% C.I. *	B	*p*-Value	95% C.I. *	B	*p*-Value	95% C.I. *	B	*p*-Value	95% C.I. *
Age	6.5	**0.000**	3.6, 9.4	1.8	0.196	−0.9, 4.5	0.2	0.667	−0.6, 0.9	0.6	**0.022**	**0.1, 1.1**
Females (Ref. Males)	−4.2	0.904	−72.9, 64.6	39.9	0.173	−17.8, 97.5	−7.1	0.438	−25.4, 11.1	−16.2	**0.011**	−28.5, −3.8
AF (Ref. MF)	−128.4	**0.005**	−216.7, −40.2	−4.6	0.908	−82.4, 73.3	31.5	**0.009**	8.2, 54.9	−6.8	0.413	−23.3, 9.7
Cirrhosis (Ref. MF)	−62.9	0.129	−144.3, 18.6	−24.3	0.487	−93.6, 45.0	31.7	**0.004**	10.3, 53.0	−8.9	0.244	−24.2, 6.2
All other genotypes (Ref. HCV-3)	−31.2	0.424	−108.6, 46.1	−15.8	0.630	−81.0, 49.3	7.4	0.507	−14.6, 29.4	−3.2	0.674	−18.1, 11.8
High VRL (Ref. Low VRL)	−22.8	0.506	−90.6, 45.1	72.6	**0.013**	15.6, 129.7	0.6	0.948	−17.4, 18.6	−15.0	**0.016**	−27.1, −2.8
BT levels	-	-	-	0.6	**0.000**	0.4, 0.8	-	-	-	0.8	**0.000**	0.6, 0.9

Key: R^2^ = adjusted R square; C.I. = confidence intervals; * = lower bound, upper bound; Ref. = reference group; AF = advanced fibrosis; MF = mild fibrosis. Statistically significant associations are presented in bold.

## Data Availability

Not applicable.

## Supplementary Materials

The following are available online at https://www.mdpi.com/article/10.3390/ijms22157961/s1.

## Appendix A

### Appendix A.1. ELISA Validation

In order to validate the ELISA immunoassays presented in the Materials and Methods, we calculated the intra- and inter-assay percentages of the coefficients of variation (CVs) (%). The intra-assay CV(%) for both ANGPTL-3 and -4 ELISAs did not exceed 7%. For the intra-day inter-assay validation, 15 serum samples were run in three ELISA plates, providing an inter-assay CV(%) that did not exceed 11% and 9% for ANGPTL-3 and ANGPTL-4, respectively. For the reproducibility between assays performed on different days, the inter-assay CV(%) was calculated by measuring 10 serum samples (at least 3 measurements per sample), and it did not exceed 14% for the ANGPTL-3 ELISA (with two outliers), whereas it was less than 10% for the ANGPTL-4 ELISA. Finally, linearity-of-dilutions experiments determined the linearity of the assays between 100- and 400-fold for ANGPTL-3 and 10- and 40-fold for ANGPTL-4, while lower dilution factors provided inconsistent lower ANGPTL concentrations in the diluted clinical samples. Spike-and-recovery assays using recombinant ANGPTLs reconfirmed the established linearity ranges.

The intra-assay CV(%), intra-day inter-assay CV(%) and inter-assay CV(%) for the TGF-β ELISA were measured at 6.5%, 10.5% and 13.3%, respectively.

### Appendix A.2. Alterations in ANGPTL Expression Levels Depend on Fibrosis Stage Following DAA Treatment

We calculated the numbers of patients who had reduced or raised ANGPTL levels per liver disease stage, following HCV eradication. For this analysis, we arbitrarily included all values that equalled or surpassed the intra-assay CV(%) threshold of 7%. As shown in Table A1, about 54% of the advanced fibrosis patients sustained similar or lower ANGPTL-3 levels than before treatment. Accordingly, more than half of the cirrhotic patients retained or increased their pre-treatment serum ANGPTL-3 concentration, thereby resisting the virus eradication-related “adjustments” of ANGPTL-3 expression. However, HCV patients with mild fibrosis exhibited the tendency to reduce their ANGPTL-3 levels three times more often than further increasing them following antiviral treatment. On the other hand, ANGPTL-4 levels were 2.5–7-fold more likely to be decreased in the serum of HCV patients following DAA treatment, depending on the disease stage during which the corresponding patients received treatment (see Table A1). This behaviour could be attributed to the “corrective” impact of the treatment on the increasing trend of ANGPTL-4 levels recorded over the course of the 30 year-long progression of HCV-induced liver disease to all stages of disease.

**Table A1:** Alteration in ANGPTL-3 and ANGPTL-4 serum levels of HCV patients after treatment (AT) depends on fibrosis stage upon DAA administration.

Disease Stage	% HCV Patients with Altered ANGPTL-3 Levels AT			% HCV Patients with Altered ANGPTL-4 Levels AT		
	Decreased	Increased	*p*-Value	Decreased	Increased	*p*-Value
Mild Fibrosis	76.3	23.7	0.001	71.4	28.6	0.011
Advanced Fibrosis	53.8	46.2	0.782	87.5	12.5	0.003
Cirrhosis	59.1	40.9	0.394	70.8	29.2	0.041

**Table A2:** Study demographics by gender.

Clinical Parameter	Male		Female	
	Mean ± SD			*p*-Value
Age	51 ± 14		54 ± 13	0.317
ALT (IU/mL)	64 ± 57		129 ± 69	0.014
**Clinical Characteristic**	***n* * (%)**	***p*-value**	***n* * (%)**	***p*-value**
VRL-low (<1 × 10^6^ IU/mL)	21 (30.4%)	0.02	35 (48.6%)	0.808
VRL-high (≥1 × 10^6^ IU/mL)	39 (56.5%)		33 (45.8%)	
Not determined	9 (13.1%)		4 (5.6%)	
Genotype		0.07		0.339
HCV1a	10 (14.5%)		15 (20.9%)	
HCV1b	13 (18.8%)		20 (27.8%)	
HCV2a	6 (8.7%)		9 (12.5%)	
HCV3	21 (30.5%)		14 (19.4%)	
HCV4	13 (18.8%)		14 (19.4%)	
Not determined	6 (8.7%)		-	

*: Number of participants as described in the main text, VRL: viral load.
