# Supplementary material for: Differential Expression of the Host Lipid Regulators ANGPTL-3 and ANGPTL-4 in HCV Infection and Treatment
Source: Int J Mol Sci. 2021 Jul 26;22(15):7961. doi: 10.3390/ijms22157961 (PMC8348577; doi:10.3390/ijms22157961)
Supplement: Supplementary file 1 [file ijms-22-07961-s001.zip › Supplementary Figure Legends-Proofs.pdf]

Supplementary Figure Legends

Supplementary Figure S1. Constitutive mRNA expression of ANGPTL-3 and ANGPTL-4 in mock-infected Huh7.5 cells. Mock-infected Huh7.5 cultures were treated similarly to the HCV-infected cultures and cells harvested at the stated time points were subjected to RT-qPCR with specific ANGPTL-3 (a) or ANGPTL-4 (b) oligonucleotide primers. The relative mRNA expression at 6h (black bar) was arbitrarily set as 1 and all other values were represented as ratio of this (grey bars). Statistically significant differences within groups are denoted by one star (p-value $\leq$ 0.05), or two stars (p-value $\leq$ 0.005).

Supplementary Figure S2. Cytotoxicity of the DAA inhibitor used in Huh7.5 cells. Huh7.5 cells were treated with either the antiviral (continuous line) or its vehicle DMSO (dotted line) for the stated time points and % cytotoxicity was calculated as described in Materials and Methods.

Supplementary Figure S3. mRNA expression of ANGPTL-3 and ANGPTL-4 in Huh7.5 cells treated with DAA *in vitro*. Huh7.5 cell cultures were treated with 3.5 μM DAA or vehicle (DMSO). The cells were harvested at the stated time points and subjected to RT-qPCR with specific ANGPTL-3 (grey bins) or ANGPTL-4 (white bins) oligonucleotide primers. The relative mRNA expression of the DMSO-treated controls were arbitrarily set as 1 (dotted line) and the ANGPTL values were represented as ratio of those.
