# Supplementary figures and images for: Differential Expression of the Host Lipid Regulators ANGPTL-3 and ANGPTL-4 in HCV Infection and Treatment
Source: Int J Mol Sci. 2021 Jul 26;22(15):7961. doi: 10.3390/ijms22157961 (PMC8348577; doi:10.3390/ijms22157961)

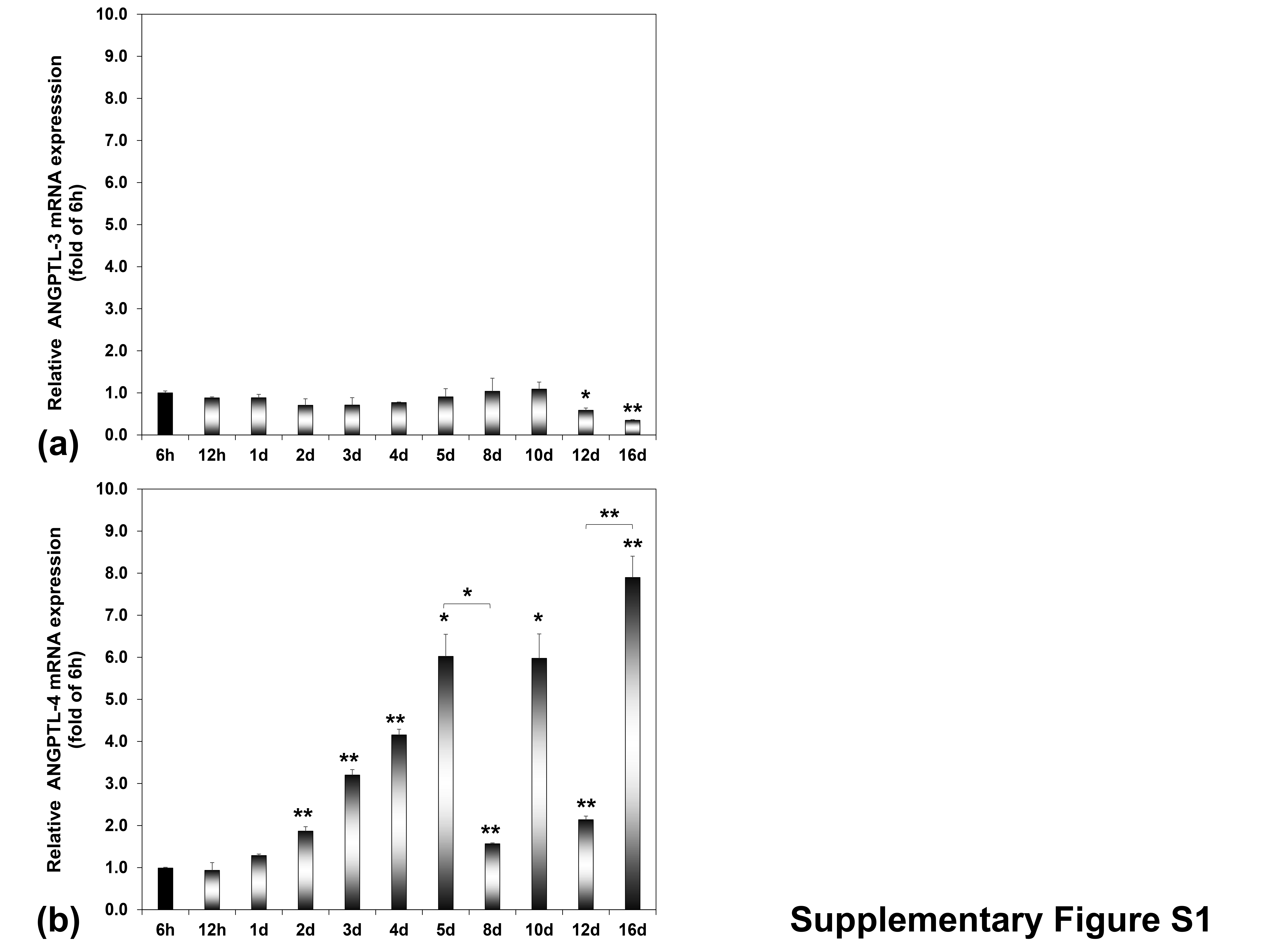

Supplement: Supplementary file 1 [file ijms-22-07961-s001.zip › Supplementary Figure S1.tif]

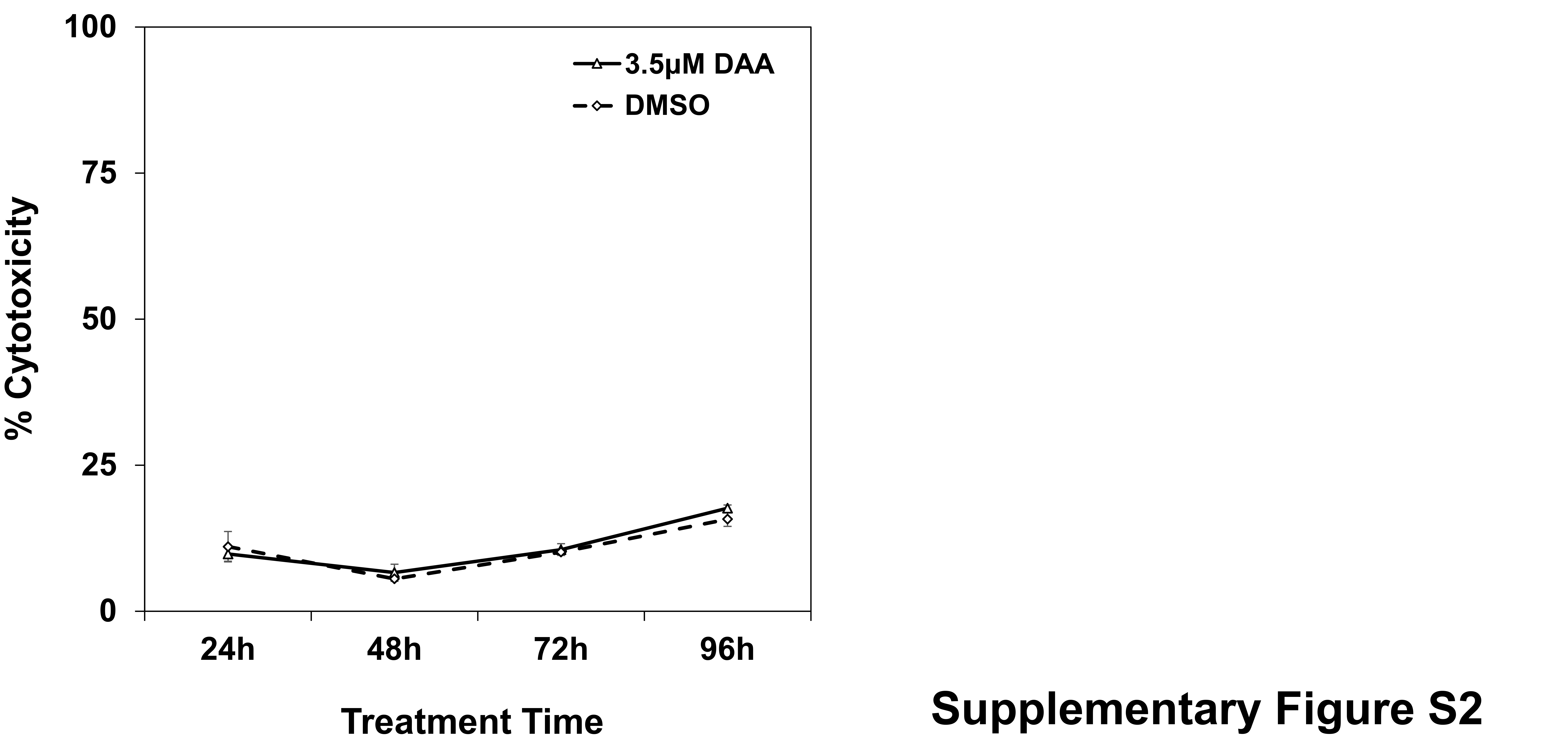

Supplement: Supplementary file 1 [file ijms-22-07961-s001.zip › Supplementary Figure S2.tif]

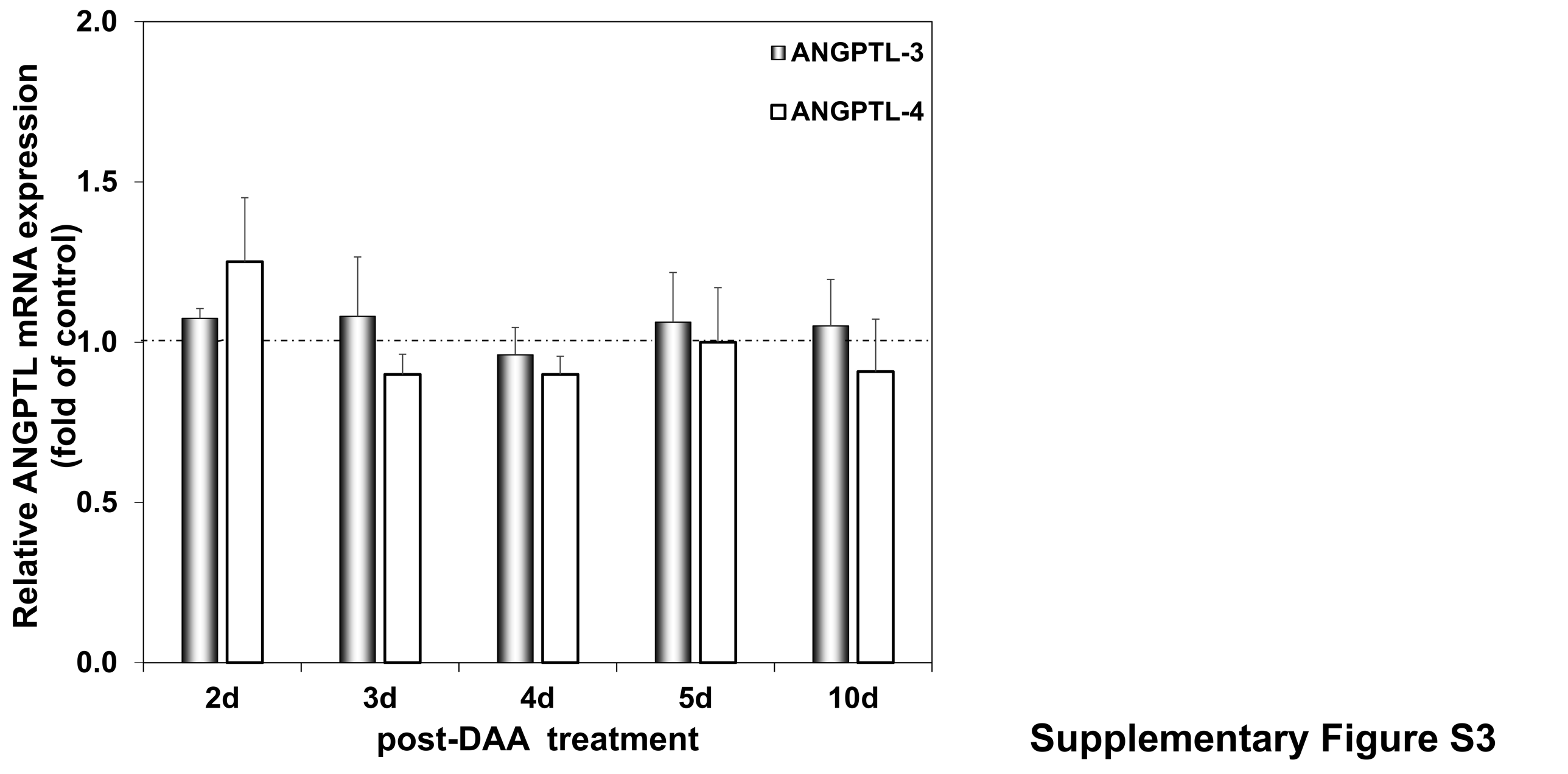

Supplement: Supplementary file 1 [file ijms-22-07961-s001.zip › Supplementary Figure S3.tif]
